# Inhibition of TGFβ1 activation prevents radiation‐induced lung fibrosis

**DOI:** 10.1002/ctm2.1546

**Published:** 2024-01-18

**Authors:** Minxiao Yi, Ye Yuan, Li Ma, Long Li, Wan Qin, Bili Wu, Bolong Zheng, Xin Liao, Guangyuan Hu, Bo Liu

**Affiliations:** ^1^ Department of Oncology Tongji Hospital Tongji Medical College Huazhong University of Science and Technology Wuhan China; ^2^ School of Computer Science and Technology Huazhong University of Science and Technology Wuhan China; ^3^ Department of Integrative Medicine Tongji Hospital Tongji Medical College Huazhong University of Science and Technology Wuhan China

**Keywords:** cilengitide, radiation‐induced pulmonary fibrosis, transforming growth factor beta 1, αv integrin

## Abstract

**Background:**

Radiotherapy is the main treatment modality for thoracic tumours, but it may induce pulmonary fibrosis. Currently, the pathogenesis of radiation‐induced pulmonary fibrosis (RIPF) is unclear, and effective treatments are lacking. Transforming growth factor beta 1 (TGFβ1) plays a central role in RIPF. We found that activated TGFβ1 had better performance for radiation pneumonitis (RP) risk prediction by detecting activated and total TGFβ1 levels in patient serum. αv integrin plays key roles in TGFβ1 activation, but the role of αv integrin‐mediated TGFβ1 activation in RIPF is unclear. Here, we investigated the role of αv integrin‐mediated TGFβ1 activation in RIPF and the application of the integrin antagonist cilengitide to prevent RIPF.

**Methods:**

*Itgav^loxP/loxP^;Pdgfrb‐Cre* mice were generated by conditionally knocking out *Itgav* in myofibroblasts, and wild‐type mice were treated with cilengitide or placebo. All mice received 16 Gy of radiation or underwent a sham radiation procedure. Lung fibrosis was measured by a modified Ashcroft score and microcomputed tomography (CT). An enzyme‐linked immunosorbent assay (ELISA) was used to measure the serum TGFβ1 concentration, and total Smad2/3 and p‐Smad2/3 levels were determined via Western blotting.

**Results:**

Conditional *Itgav* knockout significantly attenuated RIPF (*p* < .01). Hounsfield units (HUs) in the lungs were reduced in the knockout mice compared with the control mice (*p* < .001). Conditional *Itgav* knockout decreased active TGFβ1 secretion and inhibited fibroblast p‐Smad2/3 expression. Exogenous active TGFβ1, but not latent TGFβ1, reversed these reductions. Furthermore, cilengitide treatment elicited similar results and prevented RIPF.

**Conclusions:**

The present study revealed that conditional *Itgav* knockout and cilengitide treatment both significantly attenuated RIPF in mice by inhibiting αv integrin‐mediated TGFβ1 activation.

**Highlights:**

- Activated TGFβ1 has a superior capacity in predicting radiation pneumonitis (RP) risk and plays a vital role in the development of radiation‐induced pulmonary fibrosis (RIPF).
- Conditional knock out *Itgav* in myofibroblasts prevented mice from developing RIPF.
- Cilengitide alleviated the development of RIPF by inhibiting αv integrin‐mediated TGFβ1 activation and may be used in targeted approaches for preventing RIPF.

## Graphical abstract

Conditional knocking‐out *Itgav* in myofibroblasts prevented mice from developing RIPF.

Cilengitide alleviated the development of RIPF by inhibiting αv integrin‐mediated TGFβ1 activation and may be used in targeted approaches for RIPF prevention.

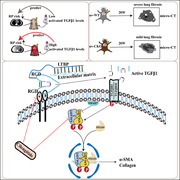

## 1 INTRODUCTION

Radiation‐induced pulmonary fibrosis (RIPF) is a common complication of chest radiotherapy and restricts the dose of radiotherapy administered.^1^, ^2^ However, the mechanism of RIPF is poorly understood, and preventive approaches and effective treatments are limited.

Although the underlying mechanism is unclear, transforming growth factor beta 1 (TGFβ1) is a pivotal mediator and central profibrotic cytokine in multiorgan fibrosis.^3^, ^4^, ^5^ The TGFβ1 gene encodes the TGFβ1 sequence in the C‐terminal region and a functional latency‐associated peptide (LAP) sequence in the N‐terminal region. Secreted TGFβ1 contains the LAP sequence and is stored in the extracellular matrix (ECM) in an inactive form. After cleavage of LAP from TGFβ1‐LAP, TGFβ1 becomes activated and exerts its biological function.^6^ TGFβ1 activators, including thrombospondin‐1, reactive oxygen species, mechanical tissue stretch and integrins, interact with the C‐terminal arginine‐glycine‐aspartic acid (RGD) sequence of TGFβ1‐LAP to degrade LAP and activate TGFβ1.^7^, ^8^, ^9^

Among the above‐mentioned activators, integrins play a critical role in mediating TGFβ1 activation in pulmonary fibrosis. Integrins are heterodimers comprising α and β subunits; 18 α subunits and eight β subunits constitute 24 integrins.^10^ Researchers previously showed that therapeutic delivery of an αvβ1 inhibitor attenuates bleomycin‐induced pulmonary fibrosis^11^ and that anti‐αvβ6 therapy protects mice against RIPF by inhibiting TGFβ1 activation but does not protect against radiation‐induced mortality.^12^

Among the integrin subunits, the αv subunit is particularly important in TGFβ1‐mediated fibrosis. Recent studies have demonstrated that knocking out αv in myofibroblasts effectively reduces TGFβ1 activation and prevents fibrosis in the liver, kidney and heart and bleomycin‐induced pulmonary fibrosis in murine models.^13^, ^14^ Myofibroblasts, which are characterised by their synthesis of ECM proteins, are pivotal cells in the pathological process of fibrosis. In vitro experiments demonstrated that myofibroblasts express αv integrin, which recognises the RGD peptide motif and activates TGFβ1.^15^ However, the role of αv integrin in RIPF has not yet been reported.

In this study, we speculated that αv integrin‐mediated TGFβ1 activation plays a crucial role in RIPF. Platelet‐derived growth factor receptor beta (PDGFRβ)^+^ cells were shown to be the primary source of lung myofibroblasts.^16^ Hence, we established *αv;Pdgfrb‐Cre* conditional knockout mice and treated wild‐type mice with the integrin antagonist cilengitide, an RGD peptide, to investigate the role of αv integrin‐mediated TGFβ1 activation in a mouse model of RIPF.

## 2 METHODS

### 2.1 Study population

A total of 44 postoperative patients with newly diagnosed stage I–IV NSCLC were included in the study. All patients received radiation therapy between March 2021 and November 2021 at Tongji Hospital, Huazhong University of Science and Technology (Wuhan, Hubei Province, China) between March 2021 and November 2021. Those eligible included patients who received 5000 cGy or more of radiation, had a Karnofsky performance status (KPS) >60, and were expected to live at least 6 months from the end of radiation treatment. Study participants with severe cardiopulmonary disorders were excluded. Approval was granted by the Tongji Hospital Review Board for the current study. Informed consent was obtained from patients before enrolment in the study.

### 2.2 Treatment and follow‐up

X‐rays delivered from a linear accelerator (Elekta Synergy) at 6 MV were used in the radiotherapy procedure. A median total dose of 5040 cGy (range: 5000–6600 cGy) was delivered at 180–200 cGy. Radiation therapy was delivered to all patients using intensity‐modulated radiation therapy (IMRT). Table 1 displays the first clinical traits and specifics of all patients' treatments.

**TABLE 1:** Patient characteristics (*n* = 44).

Characteristic	No. of patients	%
**Sex**		
Male	32	72.7
Female	12	27.3
**Age, years**		
Median	56	
Range	40–71	
**Histology**		
SCLC	6	13.6
NSCLC	38	86.4
**Stage**		
I–II	4	9.1
III–IV	40	90.9
**KPS**		
80–100	43	97.7
<80	1	2.3
**Smoking**		
Smoker	24	54.6
Nonsmoker	20	45.4
**Chemotherapy**		
Yes	11	25.0
No	33	75.0
**Radiation dose, cGy**		
Median	5040	
Range	5000–6600	
**MLD, cGy**		
Median	1049.5	
Range	614–1523.6	
**V20, %**		
Median	19.6	
Range	10.4–28.9	

Abbreviations: MLD, mean lung dose; V20, volume of normal lung receiving 20 Gy or more radiation.

All patients involved in this trial were examined prospectively weekly during radiotherapy and underwent general follow‐up examinations 1 month after radiotherapy completion, every 3 months for the first year, every 6 months for the second year, and yearly afterwards. Patients completed a history review, physical exam and computed tomography (CT) scan at each subsequent session. The radiation pneumonitis (RP) grade was assessed as previously described.^4^

### 2.3 Animals

All animal experiments were authorised by the Tongji Hospital Ethics Committee, which is connected with Tongji Medical College of Huazhong University of Science and Technology. *Itgav^loxP/−^* transgenic mice were generated in‐house in collaboration with Cyagen Biosciences, Inc., and homozygous mice (*Itgav^loxP/loxP^*) were obtained from one generation. *Pdgfrb‐Cre* transgenic mice were kindly provided by Professor Volkhard Lindner (Maine, USA). *Itgav^loxP/loxP^* mice were crossed with *Pdgfrb‐Cre* mice to generate *Itgav^loxP/loxP^;Pdgfrb‐Cre* mice. C57BL/6 mice were obtained from the Experimental Animal Center of Hubei Province (Wuhan, China). The animals were anaesthetised and given a single 16‐Gy dose for thoracic irradiation as previously described.^17^

### 2.4 Cells and reagents

MRC‐5 cells (human fetal lung fibroblasts), human bronchial epithelial (HBE) cells and human umbilical vein endothelial cells (HUVECs) were purchased from the Cell Bank, Chinese Academy of Sciences, China. MRC‐5 cells and HUVECs were cultured as described previously.^18^ HBE cells were cultured in Dulbecco's modified Eagle's medium (DMEM) supplemented with 10% fetal bovine serum (Gibco), and maintained in a humidified incubator at 37°C with 5% CO_2_. The cells were irradiated using an x‐ray irradiator (RS2000 Biological Irradiator, Rad Source). Cilengitide was purchased from GL Biochem. In the animal experiments, mice received daily intraperitoneal injections of 15 or 75 mg/kg cilengitide for 8 weeks, beginning on the day of irradiation. For in vitro experiments, the concentration of cilengitide in the cell culture medium was .5 μM prior to irradiation. Collagen content was determined using a hydroxyproline assay kit (A030‐2; Nanjing Jiancheng Bioengineering Institute). Recombinant human latent TGFβ1 protein (299‐LT‐005) and recombinant human TGFβ1 protein were purchased from R&D Systems.

### 2.5 Mouse tissue harvest and histology

Twenty‐four weeks after irradiation, blood was collected from the retro‐orbital sinuses of the mice and centrifuged at 3000 × *g* for 20 min, after which the resulting serum was stored. The mice were then sacrificed by cervical dislocation. The left lung was fixed overnight in 4% paraformaldehyde, embedded in paraffin and sectioned at 5 μm, and the right lung was used to assess protein and RNA content. Mouse lung tissue samples were stained with haematoxylin–eosin (H&E) and Masson's trichrome for histological analysis. The expression of α‐smooth muscle actin (α‐SMA) was determined using immunohistochemistry (IHC) and was analysed using a semiquantitative protocol.

### 2.6 Immunofluorescence

For immunofluorescence staining, sections stained with anti‐PDGFRβ (3169T; Cell Signalling Technology) and anti‐αv integrin (af1219‐sp; R&D) antibodies were incubated with appropriate fluorescence‐labelled secondary antibodies (sc‐362275; Santa Cruz Biotechnology; 711‐545‐152; Jackson ImmunoResearch Laboratories) and counterstained with DAPI. Images were obtained using a Carl Zeiss Axio Scope A1 microscope.

### 2.7 CT analysis of mouse lungs

Mice from each group were randomly selected and anaesthetised for thoracic CT with a micro‐CT scanner (SkyScan 1176; Bruker). Using the RadiAnt DICOM Viewer, lung density was calculated as Hounsfield units (HUs).

### 2.8 Protein extraction and Western blot analysis

Proteins were extracted from tissue samples or cultured cells, separated by electrophoresis, transferred to PVDF membranes, and hybridised overnight with the appropriate antibodies: anti‐αv integrin (611012, BD), anti‐Smad2/3 (8685T), anti‐p‐Smad2/3 (8828S), anti‐β3 integrin (4702P) and anti‐PDGFRβ (3169T) (all obtained from Cell Signalling Technology); anti‐GAPDH (AC002) and anti‐β5 integrin (A2497) (purchased from ABclonal); and anti‐α‐SMA (14395‐1‐AP) and anti‐collagen I (14695‐1‐AP) (purchased from Proteintech). Protein bands were visualised using a G:BOX Chemi X system (Syngene) according to the manufacturer's protocol.

### 2.9 Dual‐luciferase reporter assay

To confirm the effect of cilengitide on the activation of the TGFβ1/Smad signalling pathway, we utilised the pREL‐RB‐TGFβ1 signalling pathway reporter gene vector, which comprises four repeats of a Smad binding element (SBE) positioned upstream of the minimal promoter of the firefly luciferase‐encoding region (RiboBio). MRC‐5 cells were plated in 24‐well plates 24 h before transfection (1 × 10^5^ cells per well). Lipofectamine 3000 (Invitrogen) was used to transfect the reporter plasmids into cells. PBS‐ or cilengitide‐treated cells that received irradiation or underwent the sham irradiation procedure were cultured for 24 h, and TGFβ1 activity was calculated from luminescence measurements.

### 2.10 Isolation of lung PDGFRβ^+^ cells

The lungs were minced and digested in DMEM without fetal bovine serum containing type IV collagenase, hyaluronidase and DNase for 40 min. After the cells were digested, they were filtered through a 70‐μm cell strainer and lysed to obtain erythrocytes. The resuspended cells were incubated with an anti‐PDGFRβ antibody (Thermo Fisher, 12‐1402‐81, 1:200) coupled to the PE for 30 min or with a DAPI antibody (BD, 564907, 1:1000) labelled with dead cells coupled to the BV421 channel for 10 min. Finally, the PDGFRβ^+^ DAPI‐stained cells were sorted in a BD cell sorter (Sony, M6900) and analysed using FlowJo v10 software.

### 2.11 Real‐time PCR

According to previous studies, we reverse‐transcribed total mRNA from mouse lungs and cell lines into cDNA for transcriptional analysis.^18^ Real‐time PCR was performed with a 7900 HT Fast Real‐Time PCR System. A list of the primers used in this study can be found in Table S1.

### 2.12 Enzyme‐linked immunosorbent assay

One week prior to treatment (pre), as well as at 2 and 4 weeks from the start of radiotherapy, serial blood samples were taken. Blood samples were immediately placed on ice after collection. Within 4 h, the samples were centrifuged at 3000 rpm for 30 min, and the supernatants were collected and stored at −80°C until use. The appropriate number of MRC‐5 cells were seeded in 100‐mm dishes containing 16 mL of complete medium. The following day, the medium was replaced with 8 mL of serum‐free medium, and the cells were starved for 24 h, either with or without radiation treatment. The cell culture medium was concentrated to approximately 500 μL the next day. Human enzyme‐linked immunosorbent assay (ELISA) kits for the detection of TGFβ1 (ELK Biotechnology, ELK2791) and a sample activation kit (ASPEN Biotechnology, AS1240) were used to determine the concentrations of active TGFβ1 and total TGFβ1 in the plasma or cell culture medium. To prevent sample haemolysis and lessen non‐individual variations, we first extracted the same volume of blood from each mouse group and allowed it to stratify naturally for 30 min at room temperature (without shaking). The samples were centrifuged at 3000 rpm for 15 min after they had been stratified. The same volume of supernatant was taken from each mouse group, and the concentration was determined using the same standard curve to ensure quality control. Mouse ELISA kits for TNF‐α (ELK Biotechnology, ELK1387), IL‐1β (ELK Biotechnology, ELK1271), IL‐4 (ELK Biotechnology, ELK1153), IL‐6 (ELK Biotechnology, ELK1157), IL‐10 (ELK Biotechnology, ELK1143) and IL‐13 (ELK Biotechnology, ELK1145) were used to evaluate the concentrations of TNF‐α, IL‐1β, IL‐4, IL‐6, IL‐10 and IL‐13 in the plasma. Mouse TGFβ1 levels were measured using a TGFβ1 Quantikine ELISA Kit (MB100B, R&D Systems) and a sample activation kit (DY010, R&D Systems) according to the manufacturer's instructions.

### 2.13 Statistical analysis

For comparisons between two groups, Student's *t*‐test was used, and for comparisons between three groups or more, ANOVA was used. To identify significant differences between groups of mice, Kaplan–Meier survival curves were analysed using log‐rank tests. Receiver operating characteristic (ROC) curves were plotted using binary logistic regression analysis to compare the sensitivity and specificity of each factor in predicting RIPF. All the quantitative values are presented as the means ± SEMs. For all the statistical analyses, GraphPad Prism 6.0 software and SPSS 24.0 statistical software were used. *p*‐Value less than .05 was considered to indicate statistical significance.

## 3 RESULTS

### 3.1 Patient characteristics and RP

The characteristics of 44 patients, 32 males and 12 females, are shown in Table 1. The median age was 56 years (range: 40–71 years). There were 38 patients with non‐small cell lung cancer (NSCLC) and six with SCLC. Among them, 90.9% of the patients had stage III–IV disease, 25.0% of the patients received induction chemotherapy followed by radiotherapy, and all underwent surgery. The median radiation dose was 5040 cGy (range: 5000–6600 cGy), the median mean lung dose (MLD) was 1049.5 cGy (range: 614.0–1523.6 cGy) and the median V20 was 19.6% (range: 10.4%–28.9%).

The median follow‐up interval was 14 months (range: 10–19 months). Twelve patients (27.3%) exhibited grade ≥2 RP within 12 months. Table 2 lists the associations between patient‐ and therapy‐related characteristics and grade ≥2 RP. Univariate and multivariate analyses revealed that V20 and MLD were significantly related to grade ≥2 RP. Patients with V20 ≥24.0% had a greater risk of grade ≥2 RP than did those with V20 <24.0% (hazard ration [HR] = 4.209, 95% confidence interval [CI] = 1.118–15.841; *p* = .034). Additionally, patients with an MLD ≥1300 cGy had a greater risk of having a grade ≥2 RP than did those with an MLD <1300 cGy (HR = 5.15, 95% CI = 1.448–18.308; *p* = .011).

**TABLE 2:** Associations between patient‐ and therapy‐related characteristics and grade ≥2 radiation pneumonitis.

	Univariate analysis			Multivariate analysis		
Parameter	HR	95% CI	*p*	HR	95% CI	*p*
Age	2.205	.679–7.164	.188	3.049	.751–12.370	.119
Sex	1.235	.340–4.488	.749	2.063	.347–12.255	.426
Smoking	.860	.289–2.561	.787	.280	.053–1.479	.134
Radiation dose	.903	.295–2.762	.858	.533	.150–1.890	.330
MLD	4.370	1.455–13.129	.009	5.150	1.448–18.308	.011
V20	3.069	1.027–9.173	.045	4.209	1.118–15.841	.034

*Note*: Either the MLD or V20 was used in multivariate analyses, but not together.

Abbreviations: CI, confidence interval; HR, hazard ratio; MLD, mean lung dose; V20, volume of normal lung receiving 20 Gy or more radiation.

### 3.2 Activated TGFβ1 level has better RP risk prediction performance

**FIGURE 1:**
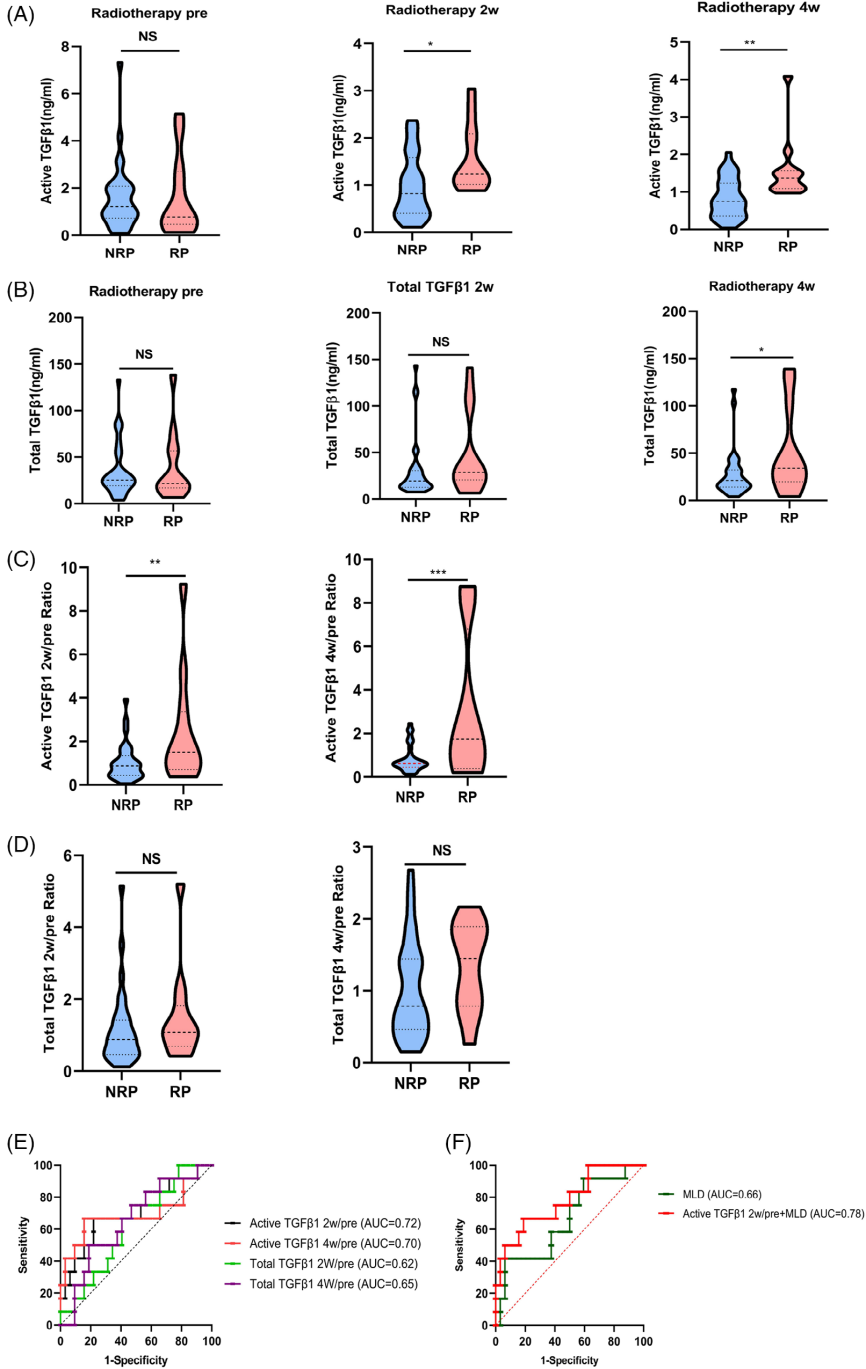
Activated TGFβ1 levels have better ability to predict radiation pneumonitis (RP) risk. The levels of activated TGFβ1 (A) and total TGFβ1 (B) in 44 postoperative patients with and without RP. (C) The activated TGFβ1 2 weeks/pre ratio and 4 weeks/pre ratio in patients with and without RP. (D) The total TGFβ1 2 weeks/pre ratio and 4 weeks/pre ratio in patients with and without RP. (E) ROC curves for the prediction of RP by activated TGFβ1 2 weeks/pre and 4 weeks/pre and total TGFβ1 2 weeks/pre and 4 weeks/pre. (F) ROC curves for the prediction of RP by activated TGFβ1 2 weeks/pre and MLD.

Neither activated TGFβ1 nor total TGFβ1 levels before radiotherapy were associated with a high risk for grade ≥2 RP. Higher activated TGFβ1 levels at both 2 and 4 weeks during radiotherapy were associated with a greater risk of grade ≥2 RP, whereas higher total levels were associated with a greater risk of grade ≥2 RP only at 4 weeks during radiotherapy (Figure 1A,B). Similarly, the activated TGFβ1 2 weeks/pre ratio and 4 weeks/pre ratio were strongly correlated with the risk for grade ≥2 RP. However, the total TGFβ1 concentration at the 2 weeks/pre ratio and 4 weeks/pre ratio did not significantly differ (Figure 1C,D). According to the ROC curve, activated TGFβ1 levels had a greater area under the curve (AUC) than total TGFβ1 levels did in predicting the risk of RP either at 2 or 4 weeks during radiotherapy. Additionally, the activated TGFβ1 2 weeks/pre ratio and 4 weeks/pre ratio displayed better specificity and sensitivity than did the total TGFβ1 2 weeks/pre ratio and 4 weeks/pre ratio (Figure 1E). The AUC was .66 for MLD, and the combination of the activated TGFβ1 2 weeks/pre ratio and MLD for predicting RP risk had the largest AUC (Figure 1F). These results suggested that it is more meaningful to measure the activated TGFβ1 level in patients than the total TGFβ1 level during radiotherapy.

### 3.3 Generation of conditional *Itgav* knockout mice

**FIGURE 2:**
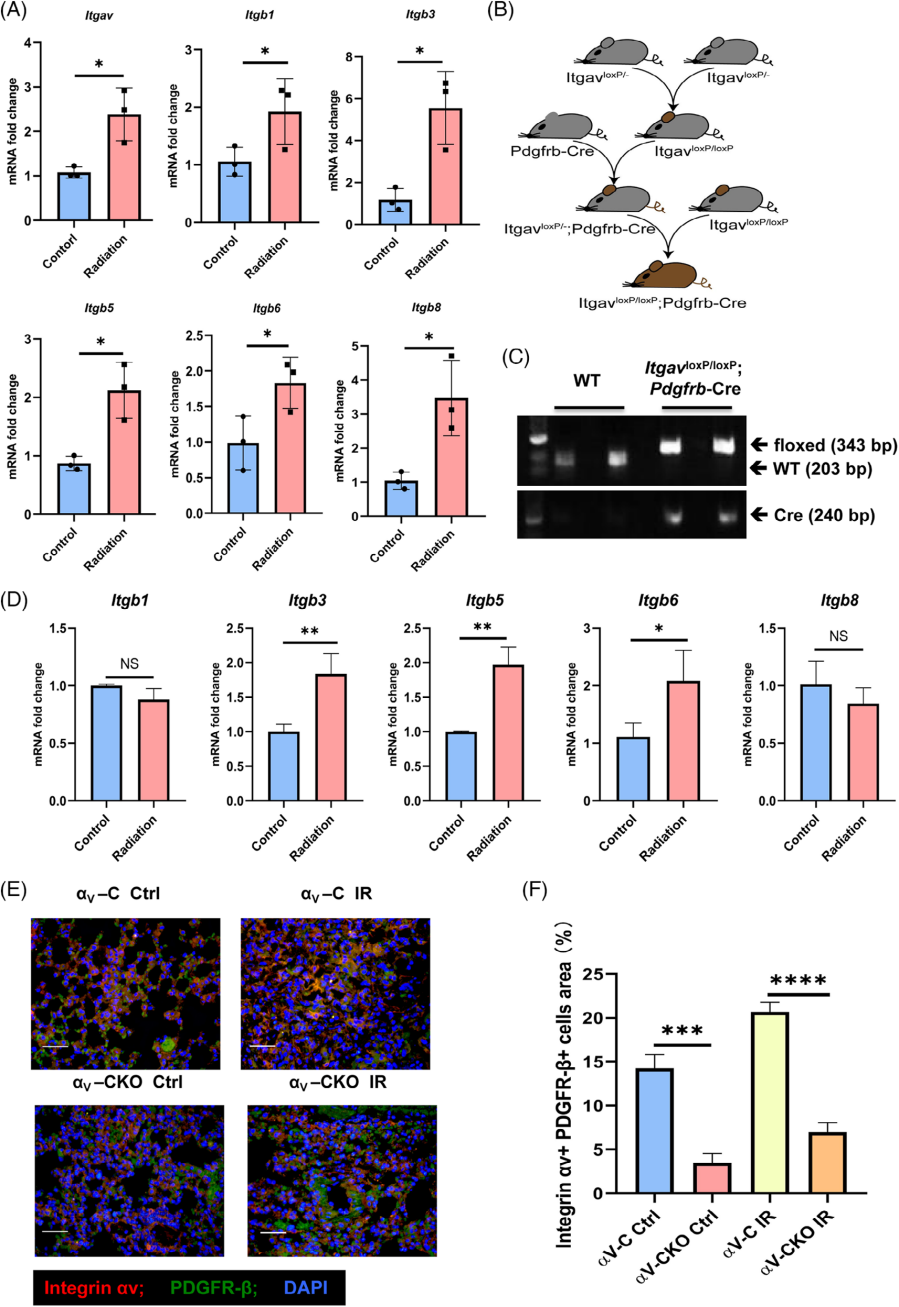
Generation of conditional *Itgav* knockout mice. (A) Real‐time qPCR analysis of αv, β1, β3, β5, β6 and β8 mRNA expression in mice at 24 weeks after irradiation or the sham irradiation procedure. (B) Schematic representation of the generation of αv‐CKO mice. (C) Representative (*n* = 9 mice per genotype) genotyping showing the WT band (203 bp) from WT mice and the αv integrin floxed band (343 bp) and Cre band (240 bp) from *Itgav^loxP/loxP^; Pdgfrb‐Cre* mice. (D) mRNA expression of *Itgb1*, *Itgb3*, *Itgb5*, *Itgb6* and *Itgb8* in PDGFRβ^+^ cells treated with IR. (E) Representative immunofluorescence images of the αv‐C and αv‐CKO mice after irradiation or the sham radiation procedure. (F) Quantitative analysis of the integrin αv^+^ PDGFRβ^+^ cell area.

To determine which integrin subunit plays an important role in RIPF, we collected lung tissue from mice 6 months after irradiation for PCR. We found that αv integrin and the αv‐associated integrins β3, β5, β6 and β8 were significantly more highly expressed in irradiated mice than in unirradiated mice (Figure 2A). Afterwards, we investigated whether αv integrin in myofibroblasts is required for the development of RIPF in vivo. We generated mice in which *Itgav* could be conditionally deleted in myofibroblasts. The mesenchymal marker PDGFRβ is specifically expressed by myofibroblasts in multiple organs, including the lungs.^19^, ^20^ We crossed mice harbouring *Itgav* flanked by *loxP* sites (*Itgav^loxP/loxP^* mice) with mice expressing Cre recombinase driven by the mouse *Pdgfrb* promoter (*Pdgfrb‐Cre* mice) (Figure 2B). Offspring that were homozygous for the floxed *Itgav* allele and hemizygous for the *Pdgfrb‐Cre* transgene (*Itgav^loxP/loxP^;Pdgfrb‐Cre* mice), as confirmed by PCR (Figure 2C), were characterised by deletion of the *Itgav* gene in myofibroblasts, and indicated successful generation of conditional *Itgav* knockout mice, herein referred to as αv‐CKO mice. Littermates were used as controls; herein, these controls are referred to as αv‐C mice. We sorted PDGFRβ^+^ cells by flow sorting from αv‐C mice and αv‐CKO mice, and the sorted images are shown in Figure S1A. PDGFRβ^+^ cells sorted from αv‐C mice were cultured, irradiated, and subsequently subjected to qRT‐PCR to detect the expression of the integrin β subunit. We found increased expression of the β3, β5 and β6 subunits after irradiation in PDGFRβ^+^ cells (Figure 2D). We performed Western blotting and qRT‐PCR after sorting PDGFRβ^+^ cells from the αv‐C mice and αv‐CKO mice by flow sorting. Our results showed that *Itgav* expressions in PDGFRβ^+^ cells from αv‐CKO mice were significantly lower than that in the av‐C mice (Figure S1B,C). The αv‐CKO and αv‐C mice were then challenged with radiation or a sham irradiation procedure. Immunofluorescence confirmed the deletion of αv integrin in PDGFRβ‐positive cells (Figure 2E,F).

### 3.4 Conditional *Itgav* knockout in myofibroblasts prevented mice from developing RIPF

**FIGURE 3:**
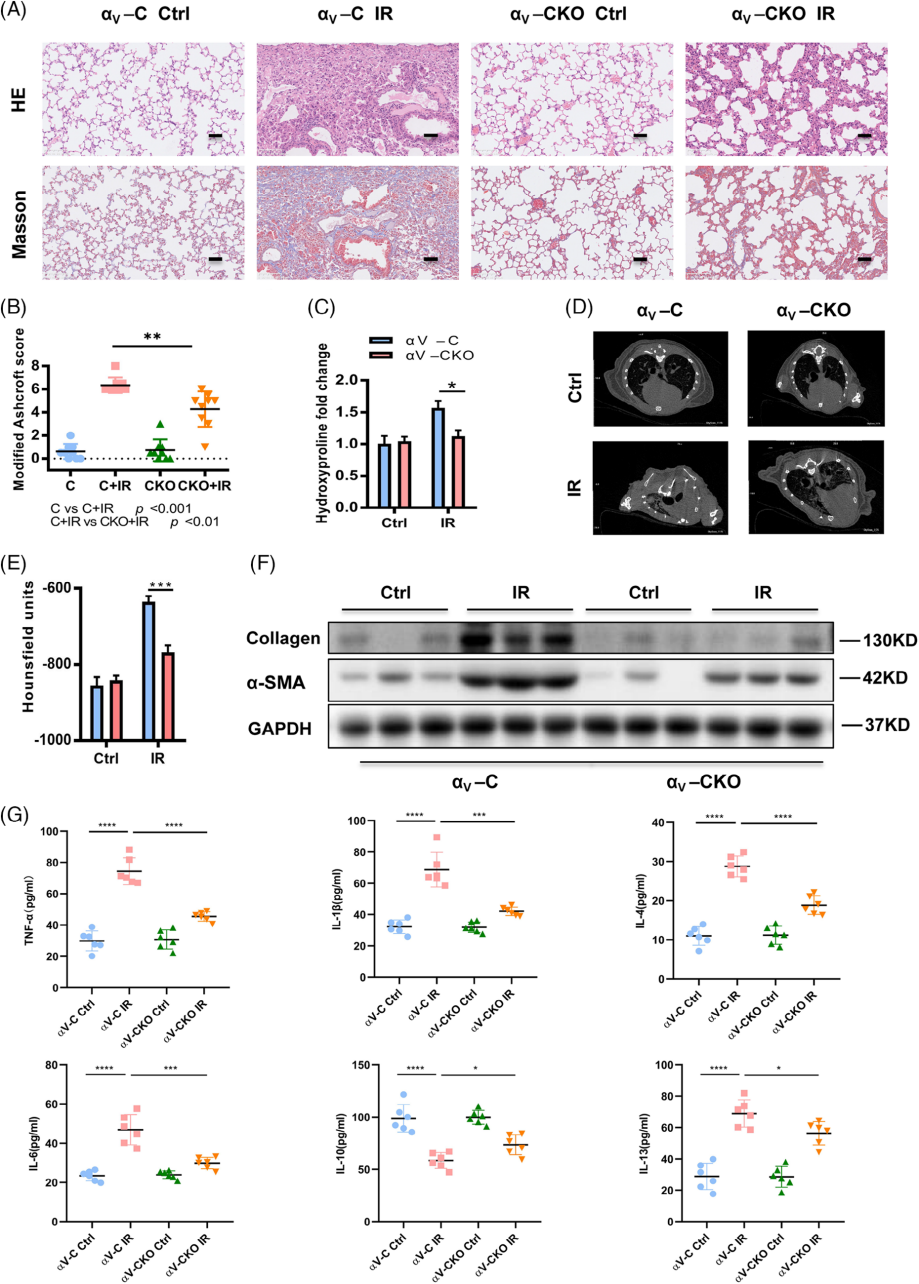
Conditional *Itgav* knockout in myofibroblasts prevented mice from developing radiation‐induced pulmonary fibrosis (RIPF). (A) Haematoxylin–eosin (H&E) and Masson's trichrome staining of lung sections from the αv‐C and αv‐CKO mice 24 weeks after irradiation or the sham radiation procedure. (B) Assessment of the severity of pulmonary fibrosis via the modified Ashcroft score. (C) Hydroxyproline levels in the lungs of the αv‐C and αv‐CKO mice at 24 weeks after irradiation or sham irradiation. *n* = 5 for all groups. (D) Representative images obtained by micro‐computed tomography (CT) after irradiation. (E) HUs derived from CT scans at 20 weeks were defined on the basis of −1000 for air and 0 for water. (F) Assessment of α‐SMA and type I collagen protein expression (normalised to GAPDH protein expression) in right lung homogenates of the αv‐C and αv‐CKO mice at 24 weeks after irradiation or the sham irradiation procedure. Bars represent the means ± SEMs (*p < .05; **p < .01; ***p < .001). (G) Cytokine levels in the serum were measured by ELISAs. Bars represent the means ± SEMs (*p < .05; **p < .01; ***p < .001).

Six months after irradiation, there was considerably less collagen deposition in the αv‐CKO lungs than in the αv‐C lungs (Figure 3A). Blinded histological analysis using the modified Ashcroft score revealed markedly less lung fibrosis in the αv‐CKO mice than in the αv‐C mice (*p* < .01) (Figure 3B), which was associated with substantially lower lung hydroxyproline (collagen) content (*p* < .05) (Figure 3C). Micro‐CT was conducted as a noninvasive method to visualise morphological changes (Figure 3D). We observed increased lung density in the irradiated αv‐C mice, while the radiological signs of fibrosis were significantly reduced in the αv‐CKO mice. HUs were measured as a quantitative assessment of lung density (*p* < .001) (Figure 3E). Furthermore, fibrotic protein marker analysis of total lung homogenates from irradiated αv‐CKO mice revealed markedly lower expression of both α‐SMA, a marker of myofibroblast activation, and type I collagen compared to that in the control mice (Figure 3F). We observed that the levels of inflammation‐related cytokines (TNF‐α, IL‐1β and IL‐6) and fibrosis‐related cytokines (IL‐4 and IL13) were significantly higher in the serum of the irradiated αv‐C mice than in that of the irradiated αv‐CKO mice. Radiation induced a decrease in the expression of the anti‐inflammatory cytokine IL‐10 in the αv‐C mice, but not in the αv‐CKO mice (Figure 3G).

### 3.5 Cilengitide protected mice from radiation‐induced mortality

**FIGURE 4:**
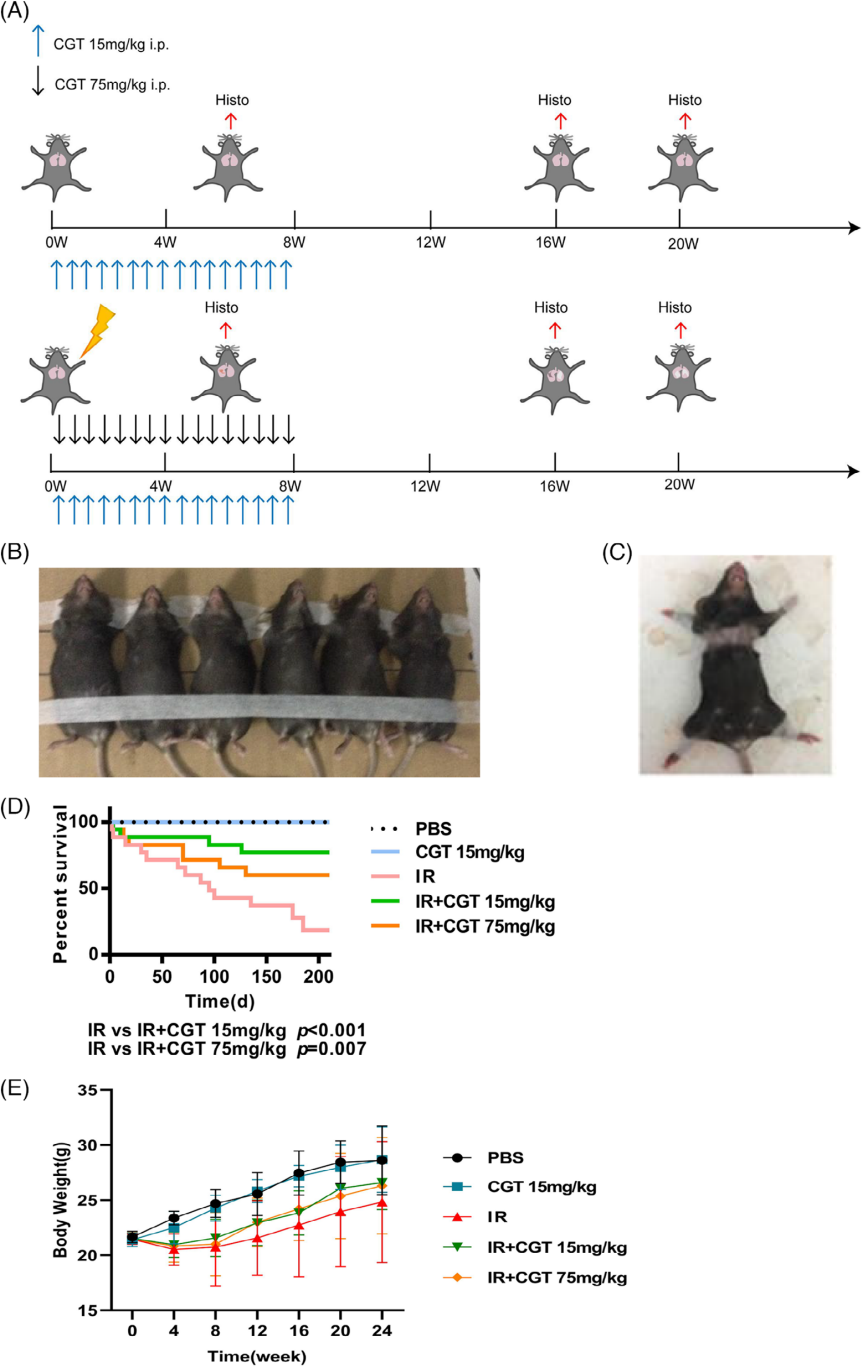
Cilengitide protected mice from radiation‐induced mortality. (A) The experimental time course starting at the time of irradiation is shown. Cilengitide was administered to the mice for 8 weeks beginning on the day the mice received 16 Gy of radiation to the thorax. The two control groups were not irradiated and received PBS or 15 mg/kg cilengitide. The other three groups were irradiated and left untreated or treated with 15 or 75 mg/kg cilengitide. (B) Photograph of mice after radiation exposure. (C) Photograph of mice at 24 weeks after irradiation. (D) Kaplan‒Meier survival curves starting at the time of irradiation were generated for 35 mice per group, and the mice sacrificed for histological examination were censored. (E) Mouse bodyweight was recorded as a general measure of health. Bars represent the means ± SEMs.

Cilengitide, a high‐affinity αvβ3 and αvβ5 integrin antagonist, prevents fibrotic progression in many fibrotic diseases by inhibiting TGFβ1 activation.^21^, ^22^ To examine the ability of cilengitide to mitigate lung fibrosis in vivo, we established a RIPF mouse model, and these mice were treated daily with cilengitide (15 or 75 mg/kg per day) or placebo via intraperitoneal injection. A schematic depiction of the mouse treatment is shown in Figure 4A. As indicated in Figure 4B, all mice were exposed to the same irradiation field. Afterwards, the mouse hair within the irradiated field turned grey during the post‐irradiation period (Figure 4C).

Mice received a single dose of 16 Gy to the thorax, leading to a significant reduction in survival compared to that of the control group (*p* < .001, log‐rank test). Cilengitide significantly attenuated this reduction in survival, especially at a concentration of 15 mg/kg, with an average survival time of 165 days, while the placebo group had an average survival time of 101 days (*p* < .001) (Figure 4D).

The adverse effects of thoracic irradiation on mice were also reflected as weight loss. While the mice from control group had a 7 g weight gain on average during the 24‐week follow‐up period, the irradiated mice gained less than .6 g. Weight loss was attenuated when 15 or 75 mg/kg cilengitide was administered after irradiation (Figure 4E).

### 3.6 Cilengitide prevented RIPF in mice

**FIGURE 5:**
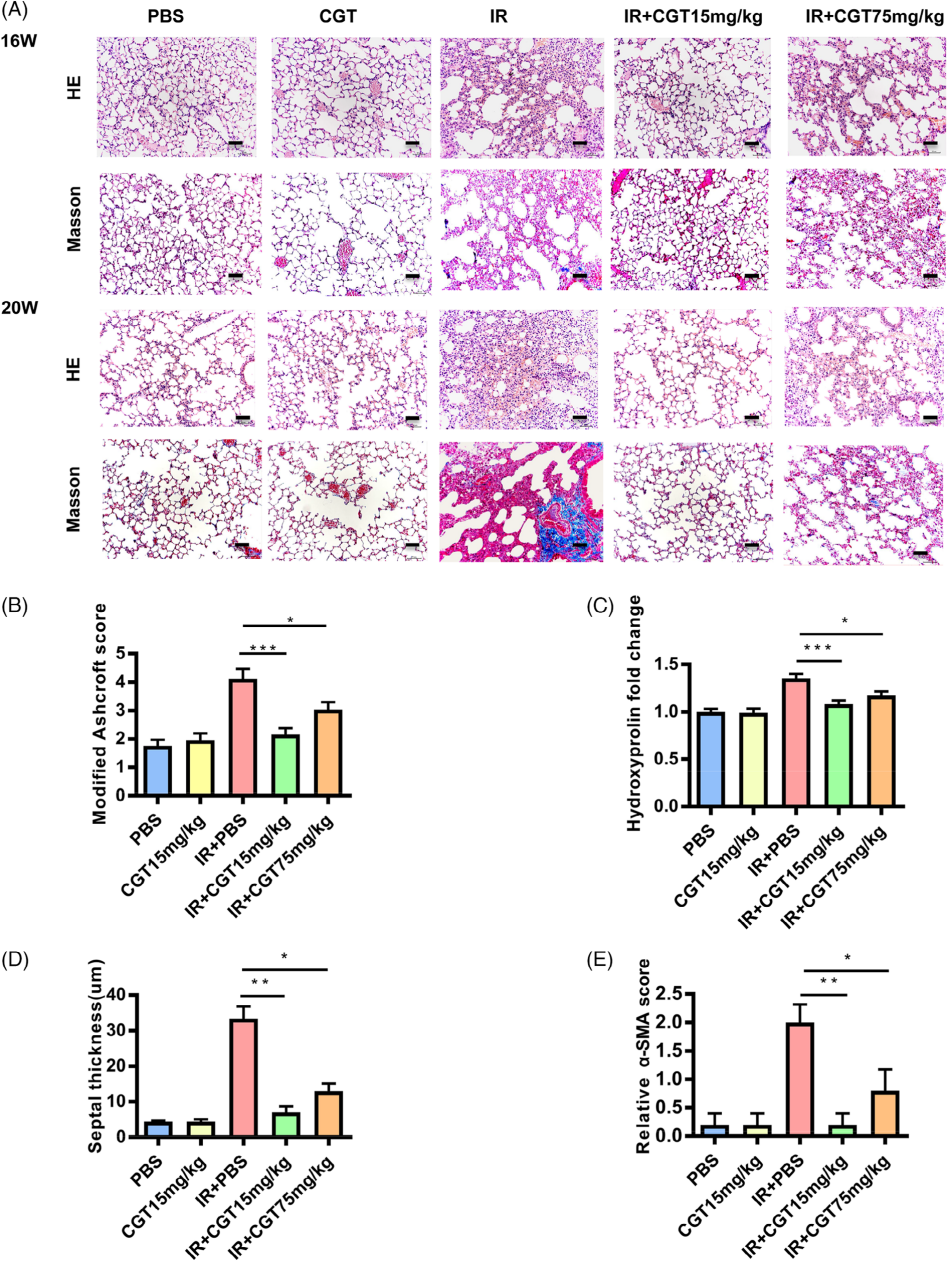
Cilengitide prevented pulmonary tissue remodelling after irradiation in mice. (A) Haematoxylin–eosin (H&E)‐ and Masson's trichrome‐stained sections from mice in each group at 16 and 20 weeks after irradiation. (B) Assessment of the severity of pulmonary fibrosis via the modified Ashcroft score. (C) Hydroxyproline content in mouse lung homogenates from mice that received irradiation and/or cilengitide at Week 20 after irradiation. (D) Average thickness of the mouse pulmonary septa after irradiation and treatment with 15 or 75 mg/kg cilengitide. (E) Real‐time qPCR analysis of *Col1a1* mRNA expression in the right lung tissue of irradiated and/or cilengitide‐treated mice. Bars represent the means ± SEMs (*p < .05; **p < .01; ***p < .001).

At 16 and 20 weeks post‐irradiation, the mice were sacrificed by cervical dislocation, and lung tissue samples were stained with H&E and Masson's trichrome (Figure 5A and Figure S2). At 16 weeks, alveolar tissue with an intact structure was rare, but fibrotic lesions were not very obvious. However, at 20 weeks after irradiation, significant collagen deposition was evident in the pulmonary interstitium. Fibrosis was significantly improved by cilengitide treatment, especially at a concentration of 15 mg/kg, as evidenced by the almost complete preservation of the mouse alveolar structure. Blinded histological analysis revealed that lung fibrosis was mitigated in the mice treated with cilengitide (*p* < .001) (Figure 5B), and this outcome was associated with a marked reduction in lung hydroxyproline levels (*p* < .001) (Figure 5C). Cilengitide treatment also resulted in an evident improvement in septal thickness (*p* < .01) (Figure 5D). Most notably, IHC analysis revealed that cilengitide inhibited the expression of α‐SMA induced by radiotherapy (Figure S3). α‐SMA expression was up to 18 times greater in the irradiated mice than in the control mice and completely returned to normal levels after cilengitide administration (*p* < .01) (Figure 5E). Picrosirius red staining analysis also showed that cilengitide significantly reduced radiation‐induced excess collagen production (Figure S4A,B). We further tested inflammation‐related cytokines (TNF‐α, IL‐1β and IL‐6) in serum samples from each group and found that TNF‐α, IL‐1β and IL‐6 were greater in the 75 mg/kg group than in the 15 mg/kg group (Figure S5). These results suggest that the use of 15 mg/kg cilengitide is more appropriate for reducing RIPF in a mouse model.

### 3.7 αv Integrin activated latent TGFβ1

**FIGURE 6:**
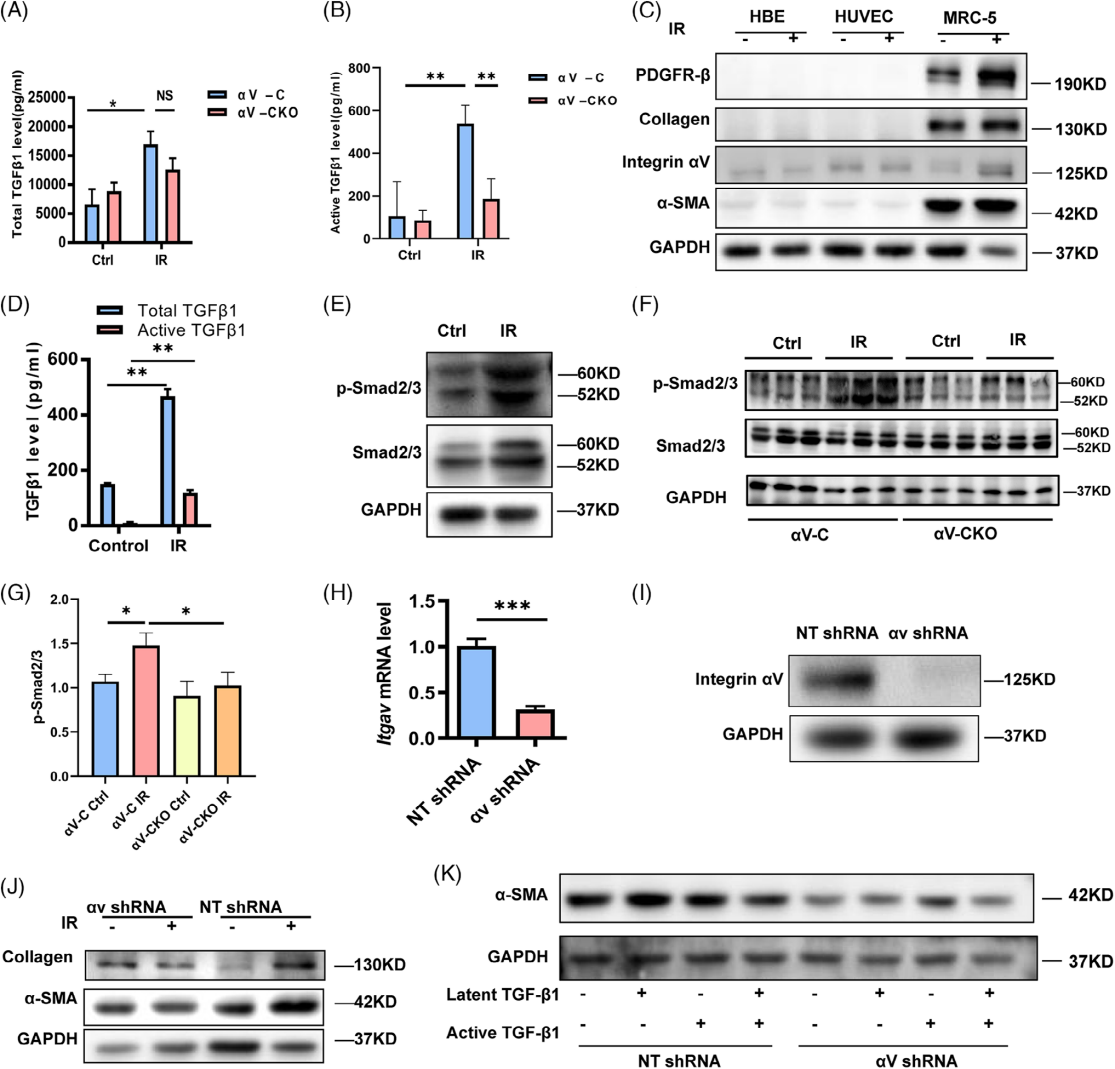
αv Integrin activated latent TGFβ1. (A and B) Total and active TGFβ1 levels in sera from the αv‐C and αv‐CKO mice after irradiation or the sham irradiation procedure were determined by ELISAs. (C) Western blot analysis of PDGFRβ, integrin αv, collagen and α‐SMA protein expression in HBE cells, HUVECs and MRC‐5 cells. (D) Total and active TGFβ1 levels in the supernatants of irradiated or sham‐irradiated MRC‐5 cells. (E) Western blot analysis of Smad2/3 and p‐Smad2/3 protein expression in irradiated (8 Gy) or sham‐irradiated MRC‐5 cells. (F) Representative Western blot images of p‐Smad2/3 and Smad2/3 in lung tissue from each group. (G) Quantitative analysis of p‐Smad2/3 in lung tissue from each group. (H) Real‐time qPCR analysis of *Itgav* mRNA levels in MRC‐5 cells transfected with NT shRNA or αv shRNA. (I and J) Western blot analysis of integrin αv, collagen and α‐SMA protein levels in MRC‐5 cells transfected with NT shRNA or αv shRNA. (K) Western blot analysis of α‐SMA expression in irradiated MRC‐5 cells treated with 10 ng/mL exogenous latent TGFβ1, 1 ng/mL active TGFβ1 or 5 ng/mL latent TGFβ1 plus .5 ng/mL active TGFβ1 and transfected with NT shRNA or αv shRNA.

We determined the serum TGFβ1 levels in the αv‐C and αv‐CKO mice after irradiation. Total TGFβ1 levels were increased after irradiation in αv‐C mice (Figure 6A), and the levels of activated TGFβ1 were lower in the αv‐CKO mice than in the αv‐C mice after irradiation (Figure 6B), suggesting that knocking out αv may affect TGFβ1 activation.

We further validated this hypothesis in vitro. We measured total and active TGFβ1 levels in cells that received 0, 2, 4, 6, 8 or 10 Gy radiation and found that cells secreted the highest levels of total and active TGFβ1 at a dose of 8 Gy (Figure S6). HBE cells, HUVECs and MRC‐5 cells were subjected to 8 Gy of radiation, and we found that only MRC‐5 cells expressed proteins associated with fibrosis, such as PDGFRβ, collagen and α‐SMA (Figure 6C). Therefore, we used the MRC‐5 cell line in all subsequent experiments. We determined the TGFβ1 content in the supernatant of MRC‐5 cells and found that both total and activated TGFβ1 levels increased after irradiation (Figure 6D), which was also consistent with the findings in PDGFRβ^+^ cells (Figure S7). Moreover, we found that the downstream protein Smad2/3 was also activated (Figure 6E). We discovered that the irradiated αv‐CKO mice had lower levels of p‐Smad2/3 than did the irradiated αv‐C mice (Figure 6F,G). We infected MRC‐5 cells with a lentivirus harbouring αv integrin‐specific shRNA or control shRNA, and the results indicated a significant reduction in αv integrin expression in the αv shRNA group compared to the control shRNA group (Figure 6H,I). After irradiation, both the collagen and α‐SMA expression levels were reduced in the cells exposed to the αv shRNA (Figure 6J). To investigate whether latent or active TGFβ1 can reverse the reduction in α‐SMA levels, we added exogenous latent or active TGFβ1 to the medium. Active TGFβ1, but not latent TGFβ1, reversed the reduction in α‐SMA expression (Figure 6K).

### 3.8 Cilengitide prevented the activation of latent TGFβ1

**FIGURE 7:**
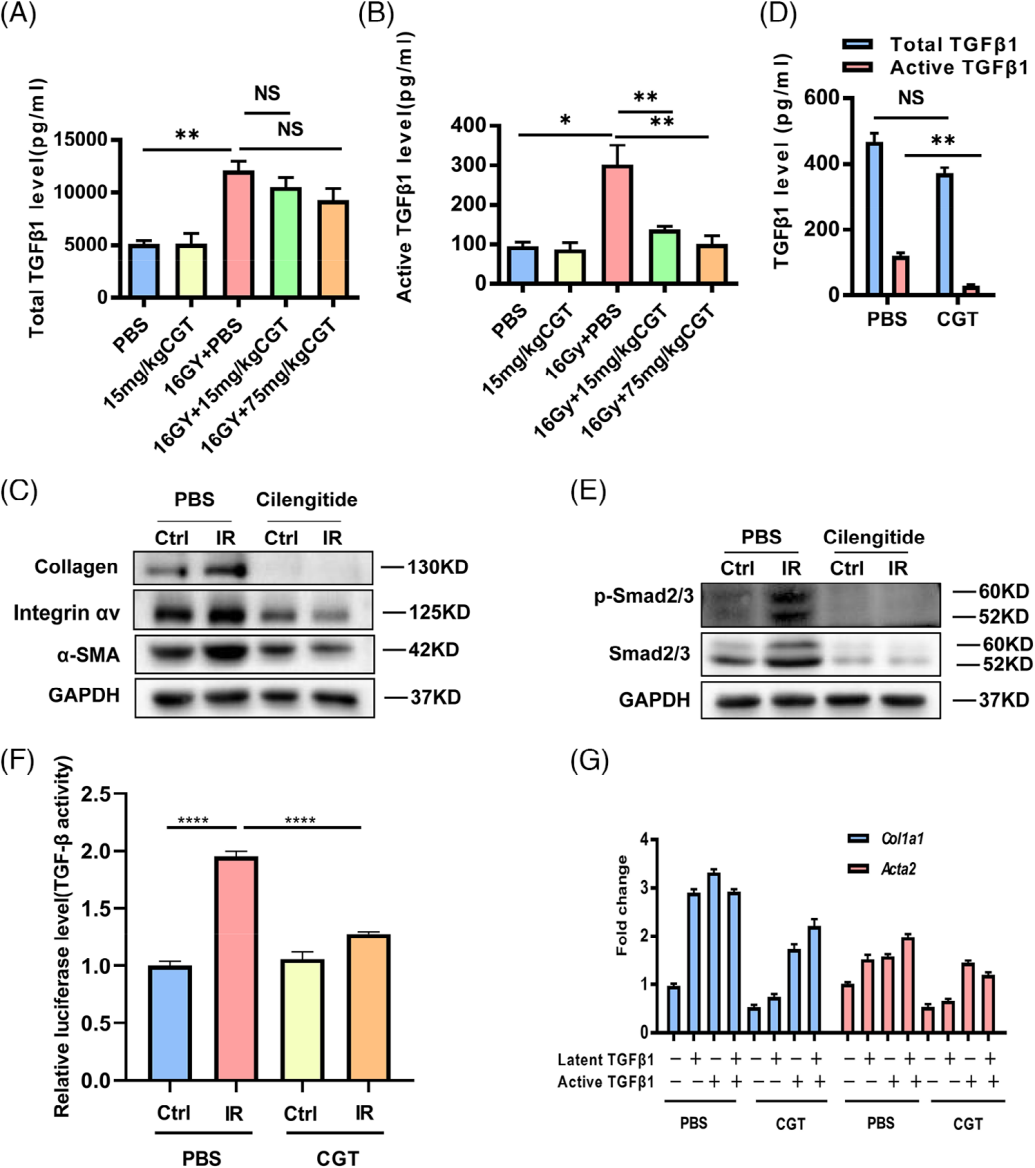
Cilengitide prevented the activation of latent TGFβ1. (A and B) Total and active TGFβ1 levels in the serum of the placebo‐ or cilengitide‐treated mice after irradiation or the sham irradiation procedure were determined by ELISAs. (C) Western blot analysis of integrin αv, collagen and α‐SMA protein levels in the PBS‐ or cilengitide‐treated MRC‐5 cells that received irradiation or underwent the sham irradiation procedure. (D) Total and active TGFβ1 levels in the supernatants of the PBS‐ or cilengitide‐treated MRC‐5 cells that received 8 Gy radiation or underwent the sham irradiation procedure. (E) Western blot analysis of Smad2/3 and p‐Smad2/3 protein levels in the PBS‐ or cilengitide‐treated MRC‐5 cells that received 8 Gy radiation or underwent the sham irradiation procedure. (F) A dual‐luciferase assay was performed to detect TGFβ1 activity. (G) Real‐time qPCR analysis of *Acta2* and *Col1a1* mRNA expression in irradiated MRC‐5 cells treated with exogenous latent TGFβ1 or active TGFβ1 and PBS or cilengitide.

**FIGURE 8:**
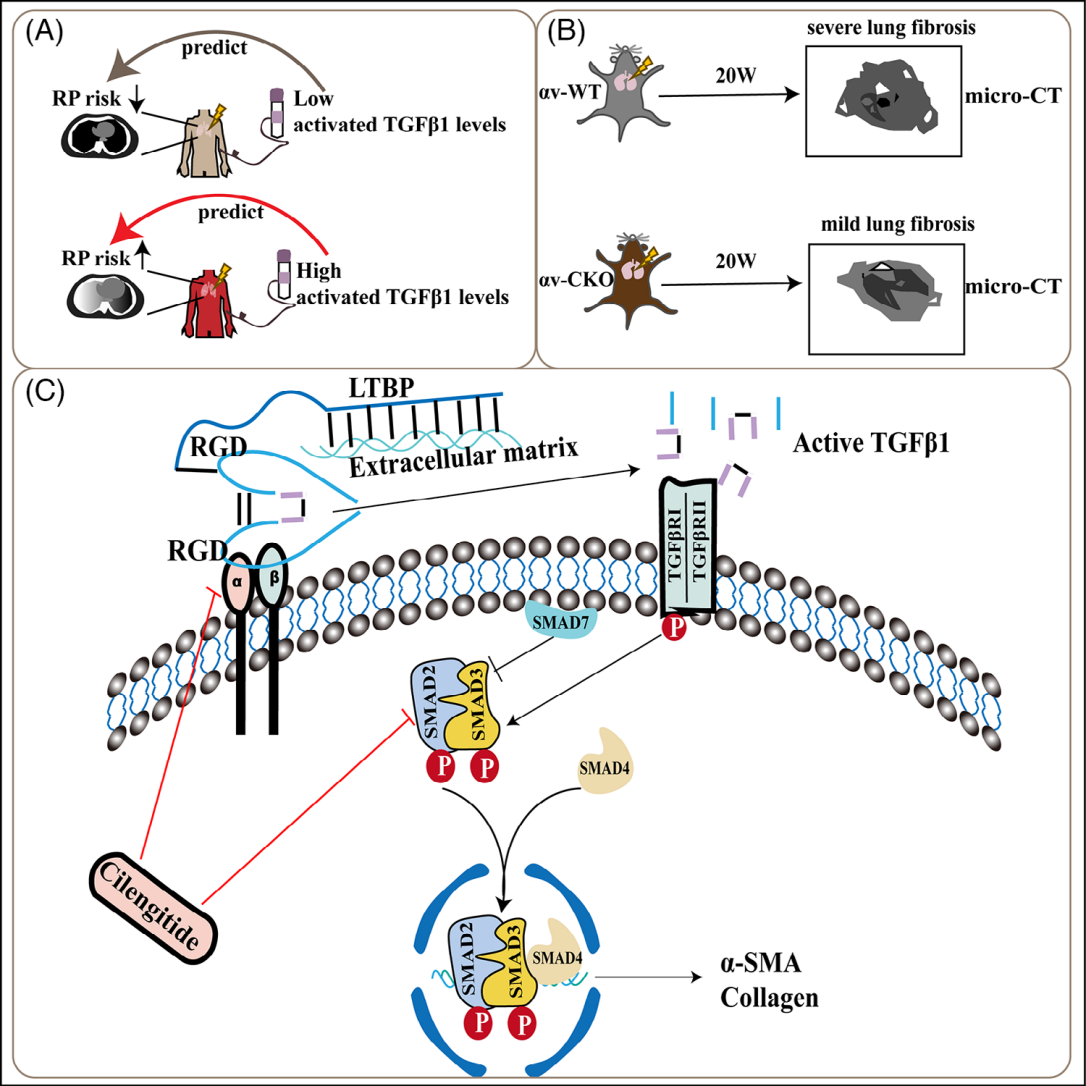
Schematic of the working model of integrin‐mediated TGFβ1 activation in myofibroblasts. (A) Activated TGFβ1 has a superior capacity in predicting radiation pneumonitis (RP) risk, and plays a vital role in the development of radiation‐induced pulmonary fibrosis (RIPF). (B) Conditional knock out *Itgav* in myofibroblasts prevented mice from developing RIPF. (C) Cilengitide alleviated the development of RIPF by inhibiting αv integrin‐mediated TGFβ1 activation and may be used in targeted approaches for preventing RIPF.

To elucidate the mechanism of cilengitide in this lung fibrosis model, we assessed the TGFβ1 concentration in mouse serum samples. Cilengitide treatment slightly reduced the total TGFβ1 concentration in the blood of the irradiated mice, but this reduction was not significant (Figure 7A); in contrast, cilengitide treatment significantly reduced active TGFβ1 levels in both the 15 and 75 mg/kg groups (Figure 7B). The proliferation of MRC‐5 cells slowed down as the concentration of cilengitide increased (Figure S8). In vitro, .5 μM cilengitide inhibited the expression of integrin, collagen and α‐SMA in irradiated MRC‐5 cells (Figure 7C). In addition, cilengitide inhibited TGFβ1 activation (Figure 7D), thus inducing the inactivation of the downstream protein Smad2/3 (Figure 7E). These findings were also confirmed in MRC5 cells stably expressing a luciferase reporter vector containing four repeats of an SBE upstream of the minimal promoter of the firefly luciferase coding region. A significant effect on reporter gene activation was observed in the irradiated MRC‐5 cells, and this effect was alleviated by cilengitide (Figure 7F). Exogenous active TGFβ1 restored the high mRNA expression levels of *Col1a1* and *Acta2*, whereas latent TGFβ1 exerted similar effects without achieving statistical significance (Figure 7G). A schematic diagram of this study is shown in Figure 8.

## 4 DISCUSSION

Research on TGFβ1 has been a hot topic in various types of pulmonary fibrosis for several years. Prior studies have noted the importance of TGFβ1 in RIPF.^4^ However, few studies have focused on activated TGFβ1 in RIPF. In the present study, we first showed that patients with grade ≥2 RP had higher levels of activated TGFβ1 at either 2 or 4 weeks from start of radiotherapy. Total TGFβ1 expression was only slightly different at 4 weeks after radiotherapy in patients with grade ≥2 RP versus those who did not develop RP. Moreover, activated TGFβ1 has superior specificity and sensitivity for predicting RP risk in patients. Therefore, the exploration of TGFβ1 activation is more clinically valuable for the prediction of RP and RIPF.

Previous studies have indicated that PDGFRβ^+^ cells are the primary source of lung myofibroblasts. PDGFRβ is expressed in diverse precursor cells of myofibroblasts, including fibroblasts, pericytes, Axin2^+^ myofibrogenic progenitors and Gli1^+^ MSC‐like cells.^16^ Therefore, we used *Itgav^loxP/loxP^; Pdgfrb‐Cre* transgenic mice to conditionally delete αv integrin in myofibroblasts to demonstrate the key role of αv integrin‐regulated TGFβ1 activation in RIPF. Under normal circumstances, radiation activates precursor cells, which then become myofibroblasts, which express integrins. On the cell membrane, integrin αv activates latent TGFβ1 in the ECM. Upon the binding of integrin to the RGD sequence in latent TGFβ1, activated TGFβ1 is released and binds to its receptor, activating the downstream Smad pathway, and thereby inducing increased expression of collagen and α‐SMA.

When *Itgav* was knocked down in myofibroblast, the cells were characterised by low α‐SMA expression. Loss of αv resulted in a decrease in secreted active TGFβ1 levels and low p‐Smad2/3 levels, suggesting that knocking down αv may inhibit myofibroblast activation by regulating TGFβ1 activation. The addition of exogenous active TGFβ1, but not latent TGFβ1, reversed these reductions, supporting the above mechanism. As described above, one potential protective mechanism resulting from the elimination or blockade of αv integrin is the inhibition of TGFβ1 activation.

RIPF is characterised by progressive and irreversible destruction of the lung structure, resulting in the loss of gas exchange function. Clinical symptoms include difficulty breathing, deterioration of lung function and accumulation of interstitial fluid, which ultimately leads to respiratory failure.^23^ Although amifostine has been approved as a radiation protectant, steroids and other forms of anti‐inflammatory therapies have been utilised to treat acute lung inflammation, but no treatment for RIPF has yet been approved for routine clinical use.^24^, ^25^, ^26^

Cilengitide, a cyclic RGD peptide, was first developed as an antiangiogenic drug to treat glioblastoma and other cancers. Recent studies have reported that cutaneous and pulmonary fibrosis are inhibited in systemic sclerosis and Crohn's disease‐induced intestinal fibrosis^21^, ^27^ and to limit the profibrotic response in cardiac fibroblasts.^28^ The pharmacological blockade of αv‐containing integrins by cilengitide inhibits TGFβ1 signalling and produces the desired antifibrotic effects without the potential side effects of blocking pan‐TGFβ1, such as autoimmunity and carcinogenesis.^7^ In our study, cilengitide reduced and prevented radiation‐induced lung remodelling and improved overall health and longevity in mice. However, one study contradicts our results; in this report, cilengitide failed to have antifibrotic effects on both thioacetamide (TAA) and bile duct ligation (BDL) models of hepatic fibrosis, but markedly increased collagen deposition and upregulated the expression of inflammatory genes, including TNFα and IL18.^29^ This discrepancy may be due to the different pathogeneses of hepatic fibrosis and RIPF. We also investigated the effect of a high dose of 75 mg/kg cilengitide on RIPF in mice. A high dose of cilengitide, although significantly inhibiting TGFβ1 activation, aggravated lung injury compared with a lower concentration of cilengitide by increasing the levels of the inflammation‐related cytokines TNF‐α, IL‐1β and IL‐6. These results suggest that the use of 15 mg/kg cilengitide is more appropriate for reducing RIPF in a mouse model.

In clinical phase 1 and 2 studies of solid tumours, cilengitide has shown good safety.^30^, ^31^ Moreover, intensive cilengitide therapy (2000 mg 5×/week during Weeks 1–6 and 2×/week thereafter) is well tolerated.^32^ These studies indicate that cilengitide possesses substantial clinical value. A recent clinical study showed that in patients with locally advanced NSCLC, cilengitide combined with chemoradiotherapy had acceptable toxicity and considerably improved survival outcomes.^33^ These studies suggest that cilengitide has good safety and effectiveness. The current and previous studies provide a basis for additional phase II clinical trials. If clinical studies further demonstrate the effectiveness of cilengitide, this drug can be used in targeted approaches for the prevention of RIPF.

Our results indicate that integrins are biologically active and regulate myofibroblast function. Therefore, strategies to disrupt myofibroblast activation by targeting integrins have potential as new therapeutic strategies for RIPF. As we discovered, treatment with cilengitide significantly reduced RIPF in mice by inhibiting TGFβ1 activation and myofibroblast activation. This study provides preclinical evidence that cilengitide can reduce RIPF.

Our research has several limitations. Although the loss of αv integrin in myofibroblasts and intervention with cilengitide provided substantial protection against RIPF, we did not observe complete protection in either model, suggesting the involvement of other mechanisms. As mentioned above, there are many other pathways that activate TGFβ1, and research on integrin‐independent pathways is lacking. In addition, we did not further block the Smad pathway to clarify its role in RIPF.

In summary, we demonstrated that conditional knockout of *Itgav* in myofibroblasts and treatment with cilengitide were both effective at preventing RIPF through the inhibition of TGFβ1 activation. We propose a new strategy for the prevention of RIPF, which could soon enter the clinical research phase and eventually be used by healthcare providers.

## AUTHOR CONTRIBUTIONS

Minxiao Yi, Ye Yuan and Li Ma contributed equally to this work.

## ETHICS STATEMENT

This study was approved by the Ethics Committee of Tongji Medical College, Huazhong University of Science and Technology. (permit number: TJ‐IRB20220321), and performed in accordance with all applicable guidelines and regulations.

## Supporting information

Supporting Information

## Data Availability

The authors will supply the relevant data in response to reasonable requests. Additional data are made available in supporting information.
