# Supplementary material for: Inhibition of TGFβ1 activation prevents radiation‐induced lung fibrosis
Source: Clin Transl Med. 2024 Jan 18;14(1):e1546. doi: 10.1002/ctm2.1546 (PMC10797247; doi:10.1002/ctm2.1546)
Supplement: Supplementary file 1 — Supporting Information [file CTM2-14-e1546-s001.docx]

**Supplementary Materials**

**Figure S1**


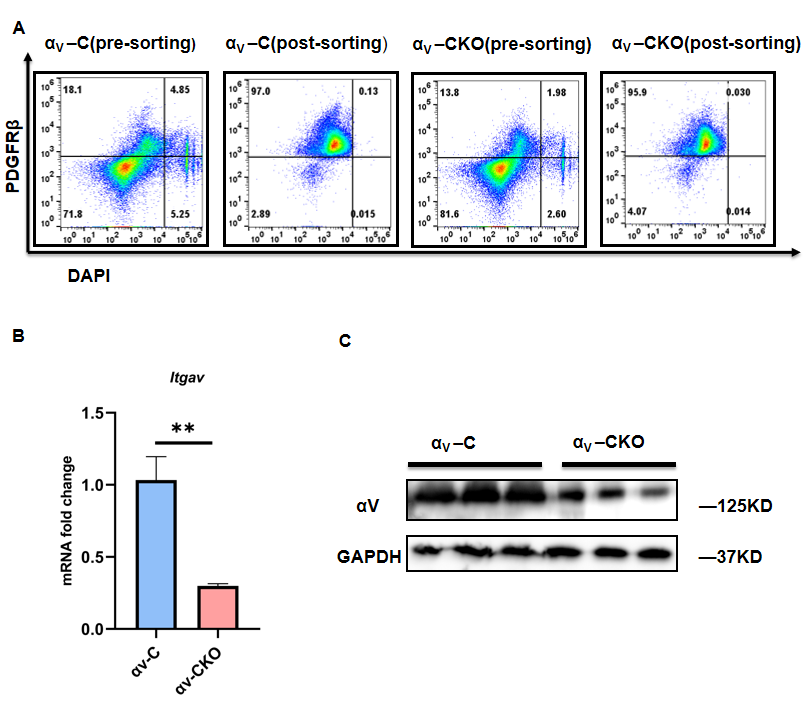


A. PDGFRβ+ cells from αv-C and av-CKO mice before and after flow sorting. B. mRNA expression of αv in PDGFRβ+ cells from αv-C and av-CKO mice. C. Protein expression of αv in PDGFRβ cells from αv-C and αv-CKO mice.

**Figure S2**


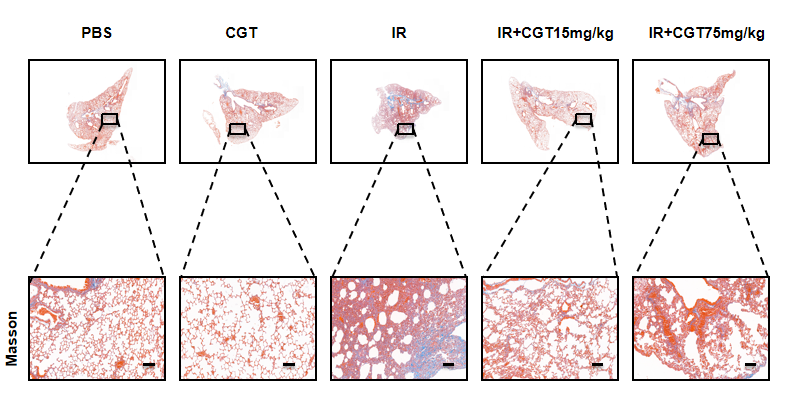


Masson’s trichrome-stained sections from mice in each group at 20 weeks after irradiation.

**Figure S3**


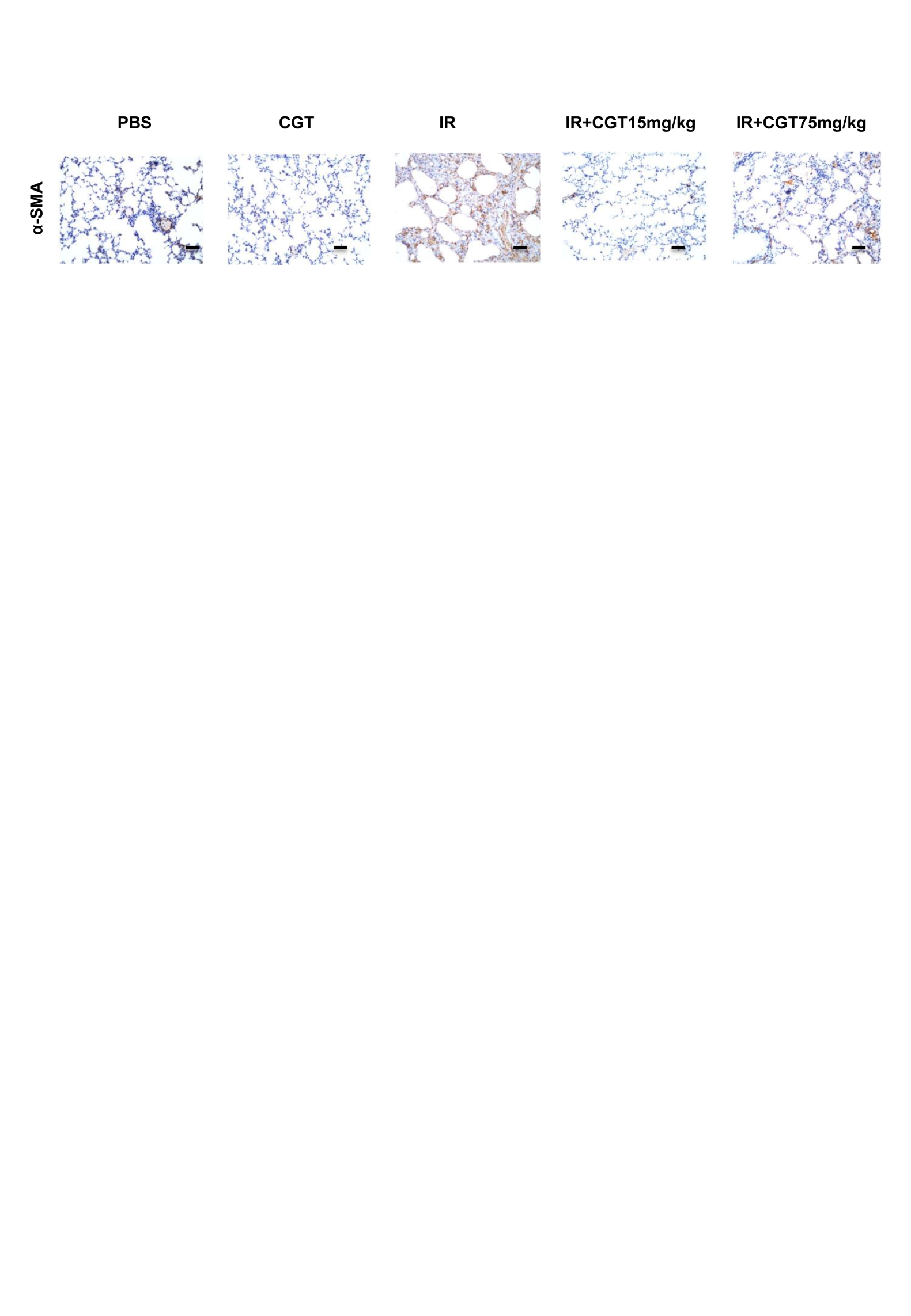


The expression of α-SMA measured by IHC.

**Figure S4**


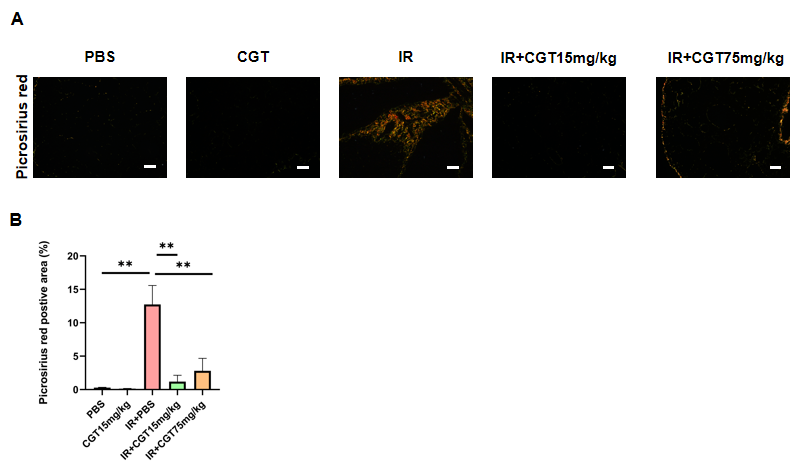


1. Picrosirius red-stained sections from mice in each group at 20 weeks after irradiation.

B. Quantitative analysis of the Picrosirius red-stained area.

**Figure S5**


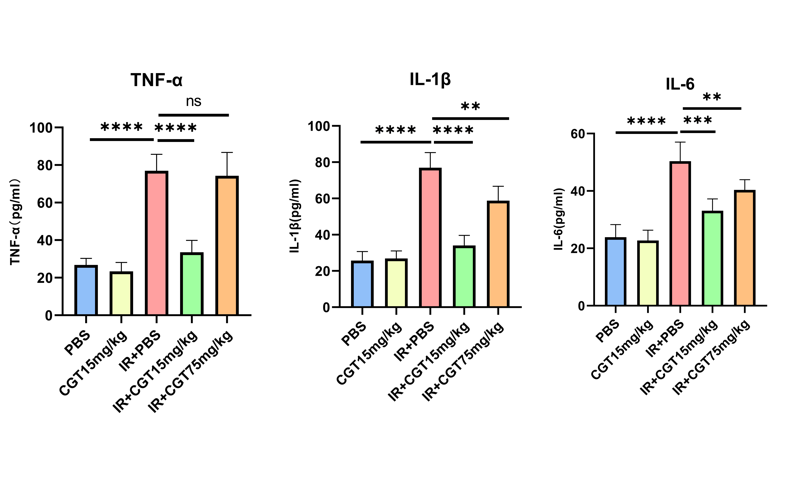


Inflammation-related cytokine (TNF-α, IL-1β, IL-6) changes in serum from each group.

**Figure S6**

Total and active TGFβ1 level in MRC-5 cells that received 0, 2, 4, 6, 8, 10 Gy.


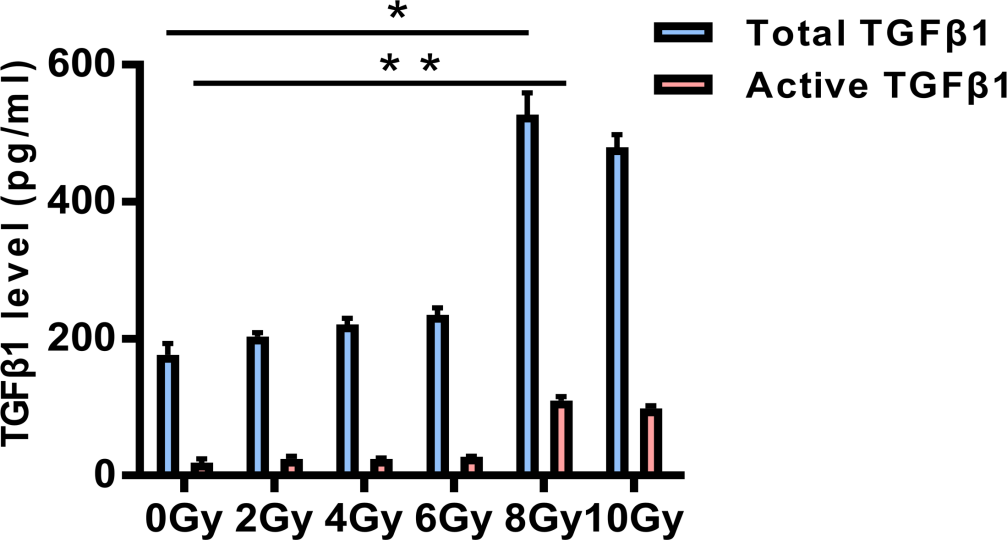


**Figure S7**

**
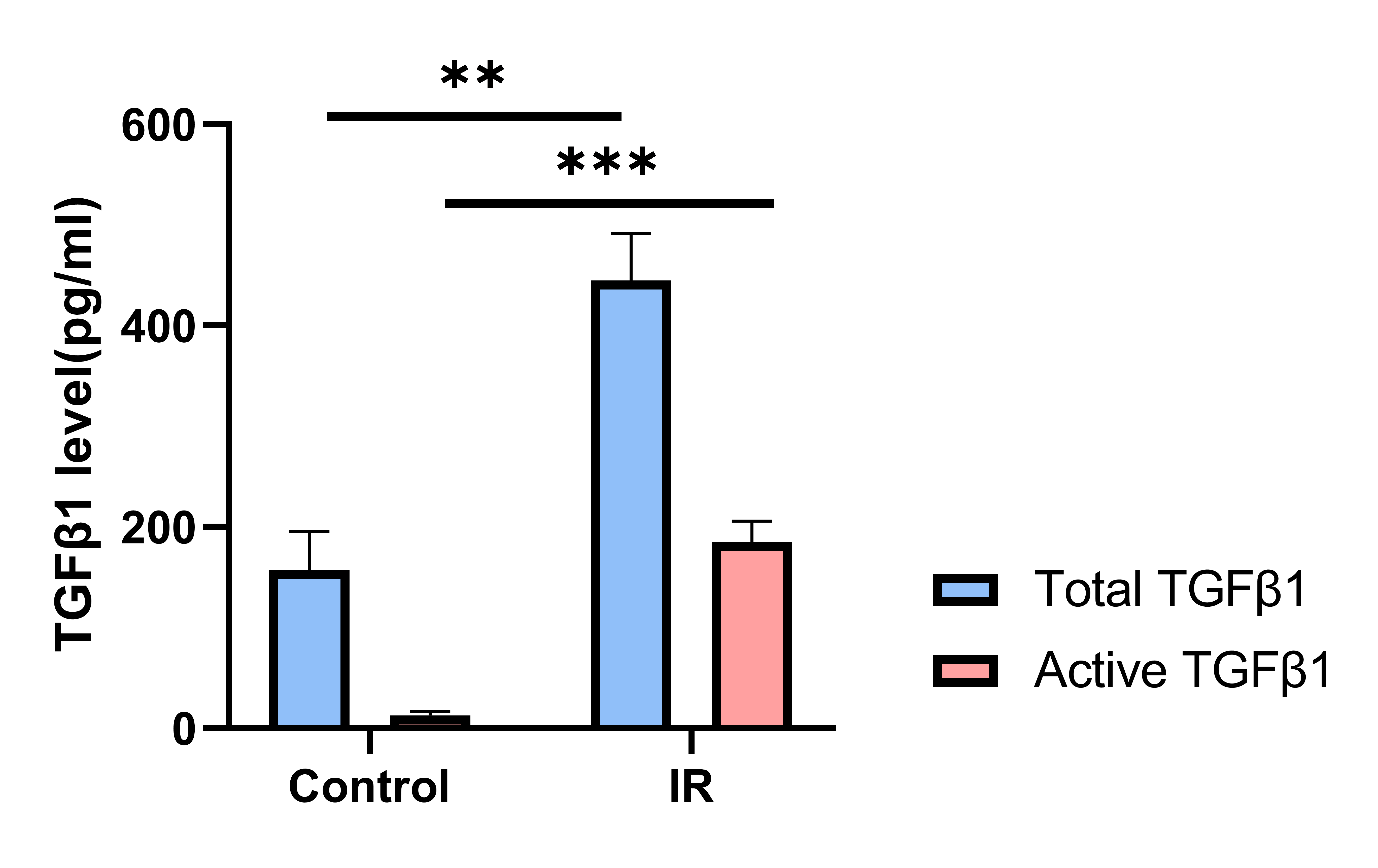
**

Total and active TGFβ1 levels in the supernatant of irradiated or sham-irradiated PDGFRβ+ cells.

**Figure S8**


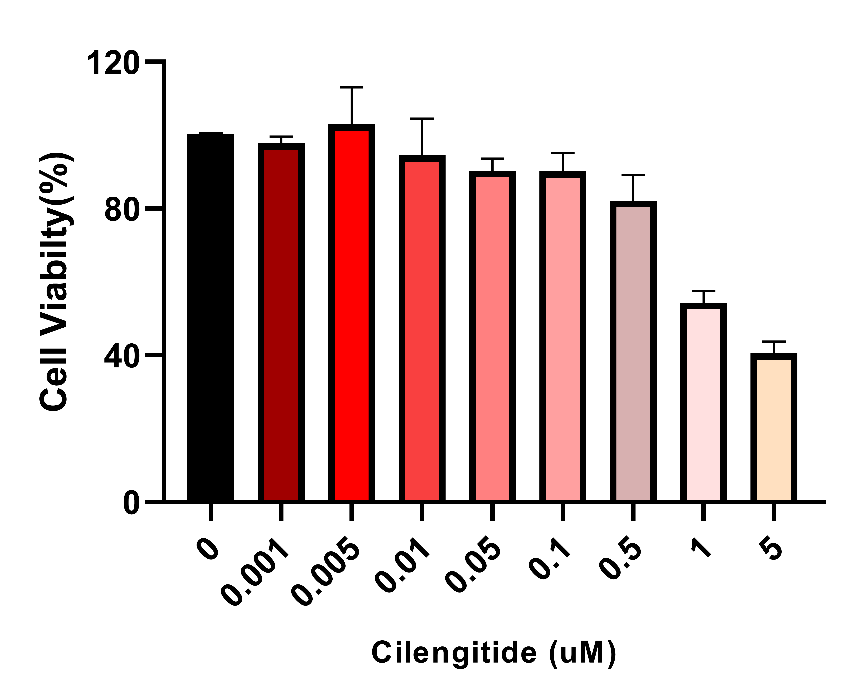


CCK-8 assay was performed to measure the sensitivity of MRC-5 cells to cilengitide.

**Table S1**

| **Gene** | **Forward Primer** | **Reverse Primer** |
| --- | --- | --- |
| *Acta2 (mouse)* | AAAAGACAGCTACGTGGGTGA | GCCATGTTCTATCGGGTACTTC |
| *Col1a1* (human) | GAGGGCCAAGACGAAGACATC | CAGATCACGTCATCGCACAAC |
| *Col1a1 (*mouse) | GCTCCTCTTAGGGGCCACT | ATTGGGGACCCTTAGGCCAT |
| *Itgav (*human*)* | ATCTGTGAGGTCGAAACAGGA | TGGAGCATACTCAACAGTCTTTG |
| *Itgav (*mouse*)* | CCGTGGACTTCTTCGAGCC | CTGTTGAATCAAACTCAATGGGC |
| *Itgb1 (*mouse*)* | ATGCCAAATCTTGCGGAGAAT | TTTGCTGCGATTGGTGACATT |
| *Itgb3 (*mouse*)* | CCACACGAGGCGTGAACTC | CTTCAGGTTACATCGGGGTGA |
| *Itgb5 (*mouse*)* | GAAGTGCCACCTCGTGTGAA | GGACCGTGGATTGCCAAAGT |
| *Itgb6 (*mouse*)* | ACTGTCTTGGTAGGTAACCTTCA | TGGCTTCATAGCAGTTGCCAC |
| *Itgb8 (*mouse*)* | AGTGAACACAATAGATGTGGCTC | TTCCTGATCCACCTGAAACAAAA |
